# *In Silico*: Where Next?

**DOI:** 10.1523/ENEURO.0131-21.2021

**Published:** 2021-04-27

**Authors:** Adrienne L. Fairhall

**Affiliations:** Department of Physiology and Biophysics, Computational Neuroscience Center, University of Washington, Seattle, WA 98195

Everyone agrees that we do not yet understand how brains work, neither well enough to satisfactorily explain basic functions such as memory nor to design effective interventions to restore mental health. This is one of the great scientific challenges of our era, with huge implications not only for human health but for insight into all animal life and for the development of future technologies. How should resources be invested to foster the necessary leap toward understanding? Given the pressing societal need and the very high public expectations of neuroscience, stoked by TED talks, New York Times articles, and sci fi, the pressures riding on choices of funding targets are enormous.

Against this background, 2013 was a banner year for the brain: both the European Union and the United States agreed to devote unprecedented support specifically to neuroscience. The European Union funded the billion-dollar Human Brain Project (HBP; Amunts et al, 2016), whose centerpiece was a team science effort to develop neuroinformatics infrastructure and to expand the Blue Brain Project (Markram, 2006), a high-fidelity biophysically realistic computational model of brain tissue. In the United States, the BRAIN Initiative launched with an initial commitment of $100 million and the appointment of a panel of respected scientists who met over months to identify major gaps and promising directions in neuroscience (Jorgenson et al., 2015). Government agencies then formulated funding opportunities that supported individual labs or small teams to develop new technologies to record, stimulate, analyze, and interpret neural activity. These efforts stimulated brain initiatives in several other countries, including Japan, Korea, and China (Huang and Luo, 2015; Grillner et al., 2016). Almost 10 years later, it is timely to look back at some of the large-scale neuroscience efforts of the past decade and the routes they have promoted toward a better understanding of the brain.

While bold in conception, the HBP was not the first Big Neuroscience effort. In 2003, Paul Allen gave $100 million to establish the Allen Institute for Brain Science. Community consultation guided this investment toward the goal of developing an information resource that would be of broad value to the community to enable and accelerate discovery: “a map of the mammalian brain at the cellular level. Through a collection of gene expression maps, brain circuitry and cell location, the [Allen Brain] Atlas will illustrate the functional anatomy of the brain” (Allen Institute Press Release, 2003; Lein et al., 2007). While inevitably imperfect, the by now seven Allen Brain Atlases (of mouse, nonhuman primate, and human brains) have been cited by thousands of papers. Paul Allen, however, wanted the Institute to go further: to contribute to the solution of a neuroscience problem. The problem chosen was vision, with a particular emphasis on the role of cell types. Launched under the leadership of Christof Koch in 2012, the MindScope project aimed “to provide a quantitative taxonomy of cell types and their interconnections in visual cortex and associated brain regions, to observe their dynamics under physiological conditions in behaving mice, to construct cellular models of how their dynamics and function arise from the structural description, and to understand how this function relates to visual perception” (Koch, 2012). This effort has resulted in the most complete characterization to date of cortical and thalamic neuronal cell types (Tasic et al., 2018), as well as a large-scale publicly available physiological database of visual responses to a battery of stimuli (de Vries, 2020), which has revealed considerable complexity. Hundreds of papers from within the Institute and dozens from outside have reported on aspects of these data, along with methodological contributions. Nonetheless, an understanding of how cells, their connections and activity patterns result in perception is still elusive.

Since these projects began, the landscape for neuroscience research has radically transformed. With help from BRAIN Initiative support (duLac, 2019), the throughput of calcium imaging has dramatically scaled up, with reports of a million simultaneously recorded neurons (Kim et al., 2016; Demas et al., 2021), the international consortium-developed Neuropixels probes are revolutionizing electrophysiology (Jun et al., 2017), a plethora of genetic mouse lines allow cell type-specific observation and manipulation (Daigle et al., 2018), and optical sensors now exist to record spatiotemporal dynamics of multiple neurotransmitter types (Sabatini and Tian, 2020). Assisted by advances in machine learning, fast tools are now available to analyze electrophysiological and calcium signals (Stringer and Pachitariu, 2019; Buccino et al, 2020), connectomic electron microscopy images (Jain et al., 2010) and behavior (Mathis and Mathis, 2020). New statistical methods are capable of extracting sophisticated dynamical models from population recordings (Pandarinath et al., 2018; Urai et al., 2021). Trained artificial neural network simulations provide an intriguing approach to developing hypotheses about the dynamics supporting neural function (Barak, 2017). BRAIN funding has thus helped to significantly raise the technological platform for discovery.

On this backdrop, what are valuable targets for future large-scale spending?

Doing good science is hard. Doing meaningful Big Science, team science at a large scale with outcomes that justify large spending, is harder. In neuroscience, unlike particle physics, there is no definitive experiment to prove or disprove an overarching fundamental theory (Frégnac, 2017). Thus, it is tempting to propose the collection of another large and complex dataset that will enable discovery, especially given advances in data collection and analysis. However, there is no agreement on a definitive dataset that will transform the field.

One current proposal is to derive the complete connectome of a mouse brain at cellular resolution (Abbott et al., 2020). Prodigious in scale, this project would take advantage of advances in tissue slicing technology and machine learning to recover the geometry and synaptic connectivity of every neuron in an individual mouse brain. Such a dataset would provide important information about circuit structure and statistical properties of connectivity, helping to constrain models (Litwin-Kumar and Turaga, 2019), but there are also major caveats (Gomez-Marin, 2021). While a detailed biophysical model without realistic connections may not provide insight into brain function, modeling based on connectivity without information about dynamical properties resulting from variations in ion channel properties, gap junctional coupling and the effects of neuromodulation, all of which can alter effective local computations, may also be severely limited.

An alternative big data approach is to record a “brain activity map” (Alivisatos et al., 2012), a survey of brain activity during a specific behavior. How should one design such a study to achieve conclusive understanding? A full understanding of any aspect of brain function should bridge multiple levels (Marr, 1982; Fairhall, 2014; Frégnac, 2017): an identification of the algorithms that govern the process, a description of how that algorithm is implemented by the hardware of neural circuitry, and a low-dimensional understanding of the resulting dynamics. This both underscores the need for, ideally, multiple competing theoretical frameworks, and also raises the question of the level of detail required in the measurements to bridge all the way to implementation. There is no single “theory of the brain” to test, so one must choose some specific subsystem, as MindScope has done with vision. Sensation, cognition and movement are all amenable to theoretical or algorithmic formulations and analysis that can predict and help to explain aspects of behavior and, to some extent, of neural processing. By selecting one function and measuring “everything possible,” one might hope to discover principles of neural organization and dynamics that generalize to other problems.

IARPA’s Machine Intelligence from Cortical Networks, or MICrONS, program (Cepelewicz, 2016) is an attempt to bridge these approaches. This ongoing $100 million project, awarded in 2016, coordinates effort of teams at Baylor, the Allen Institute, Carnegie Mellon University, Harvard, and Princeton to reconstruct the connectome of a section of visual cortex whose activity has been characterized *in vivo*. By establishing the functional relevance of specific neurons, this project addresses some of the criticisms made of both the Blue Brain (Yong, 2019) and connectomics approaches. Examining the eventual value and use of connectomic information in this targeted approach will be an informative test of whether and how detailed structural information can lead to new insights.

Another creative intermediate scale team science approach is that taken by the International Brain Laboratory (The International Brain Laboratory, 2017). Neuropixels probes record of order a thousand neurons and can only be deployed in a handful of locations in a single animal’s brain. A consortium of labs thus joined forces in 2018 to distribute the job of recording many brain areas during a standardized decision-making behavior. The project includes a computational team pursuing different aspects of analysis and theory. This heroic project is making both methodological and conceptual progress (Aguillon-Rodriguez et al, 2020; The International Brain Laboratory, 2020) but also underscores the challenges in designing a suitably complex reproducible behavior and in implementing experimental methods systematically and reproducibly in multiple labs subject to turnover of trained personnel.

Taken *in toto*, these examples illustrate several points about large scale studies. First, we see a pull toward the collection of static open datasets or development of generic large-scale models as platforms. Such a route stands in contrast to the posing and solution of specific problems. This choice is natural: such contributions can in principle benefit the community broadly and aid in the solution to many problems. In some sense they are “safe,” in that progress is quantifiable in terms of cubic millimeters or neurons measured or simulated, rather than in the more nebulous domain of questions answered. Large-scale projects that instead aim to solve one big problem are considerably more challenging: they put a much higher bar on conceptual ingenuity, have murky milestones with no clear end point and are subject to infinitely unfolding complexity. They must also lock in many experimental parameters at an early stage so that data can be collected systematically and reproducibly. This limits the feedback loop between observation and study design, reducing the ability either to change direction in the light of interesting intermediate findings, or to take rapid advantage of technological improvements. They may also run the risk of limiting a team’s ability to generate diverse ideas as the project may settle into a kind of groupthink.

What might be an alternate paradigm? The BRAIN Initiative’s key successes have arisen from the funding of many labs, distributing risk and encouraging an ongoing competition of ideas. To capitalize on these successes, a smart use of funds may be the establishment of brain observatories (Alivisatos et al., 2015; Koch and Reid, 2012). Proposed models for brain observatories would centralize large-scale systematic behavioral training, surgery, recording technology and data preprocessing but solicit proposals for behavioral paradigms and recording locations from the community. Distributed individuals or teams would then analyze and interpret the results. This honors the drive for systematic, reproducible, high quality data taking advantage of expensive cutting-edge technologies but harnesses the diverse and evolving wisdom of the community to solve brain systems. The model allows for individual innovation, ongoing evolution in questions asked, nimble experimental design, responsive testing of a wide variety of ideas, and the continuous updating of techniques. It could further support the dissemination of advanced skills and techniques back to participating laboratories, and assume the burden of open data sharing, so that each experiment would contribute to an organized, accumulating database, multiplying impact and accelerating discovery. The Allen Institute has piloted a limited version of such a scheme with its OpenScope model, which solicited vision-related proposals operating within currently implemented training and recording pipelines (Allen Institute Press Release, 2018). It is exciting to imagine the transformational impact of scaling up such a model to a wide variety of stimuli as well as behavioral and neural manipulations.

All of the scientists interviewed in Noah Hutton’s incisive documentary, *In Silico*, said: we know nothing. In fact, we know a lot, yet we still have many more questions than answers about brain function. This is inherent to the fragmented nature of what it means to “understand” the brain. We may now have reached the right time to capitalize on technological advances by creating a cutting-edge experimental platform that would allow the pursuit of many questions with the creative input of the entire field, while bringing to bear the full power of new methods, large N, reproducibility, and transparency.

## Synthesis

Reviewing Editor: Christophe Bernard, INSERM & Institut de Neurosciences des Systèmes Decisions are customarily a result of the Reviewing Editor and the peer reviewers coming together and discussing their recommendations until a consensus is reached. When revisions are invited, a fact-based synthesis statement explaining their decision and outlining what is needed to prepare a revision will be listed below. The following reviewer(s) agreed to reveal their identity: NONE.

N/A
